# Novel 2-indolinones containing a sulfonamide moiety as selective inhibitors of *candida* β-carbonic anhydrase enzyme

**DOI:** 10.1080/14756366.2018.1564045

**Published:** 2019-02-06

**Authors:** Atilla Akdemir, Andrea Angeli, Füsun Göktaş, Pınar Eraslan Elma, Nilgün Karalı, Claudiu T. Supuran

**Affiliations:** a Computer-aided drug discovery laboratory, Department of Pharmacology, Faculty of Pharmacy, Bezmialem Vakif University, Istanbul, Turkey;; b Sezione di Scienza Farmaceutiche, Neurofarba Department, Universita degli Studi di Firenze, Florence, Italy;; c Department of Pharmaceutical Chemistry, Faculty of Pharmacy, Istanbul University, Istanbul, Turkey

**Keywords:** Sulfonamides, *Candida glabrata*, carbonic anhydrase, docking

## Abstract

Inhibition of the β-carbonic anhydrase (CA, EC 4.2.1.1) from pathogenic *Candida glabrata* (CgNce103) by 1*H*-indole-2,3-dione 3-[*N*-(4-sulfamoylphenyl)thiosemicarbazones] **4a–m** was investigated. All the compounds were found to be potent inhibitors of CgNce103, with inhibition constants in the range of 6.4-63.9 nM. The 5,7-dichloro substituted derivative **4l** showed the most effective inhibition (K_I_ of 6.4 nM) as well as the highest selectivity for inhibiting CgNce103 over the cytosolic human (h) isoforms hCA I and II. A possible binding interaction of compound **4l** within the active site of CgNce103 has been proposed based on docking studies.

## 1. Introduction

*Candida* species are yeasts that normally live on human skin, mucous membranes, and the gastrointestinal tract without causing infections. However, in immunocompromised patients these microorganisms can cause fungal infections of the mouth or throat, mucous membranes or vagina (candidiadis) or it may enter the blood stream to cause more serious candidemia^1^^,^^2^. While many *Candida* species such as are responsible for these infections, *Candida glabrata* infections are becoming more frequent^3^. The development of drug resistance against the clinically used antifungals is a very important medical problem. Compared to other *Candida* strains, *C. glabrata* infections are more difficult to treat because of the rapid development of drug resistance against many classical antifungal agents^4^^,^^5^. The *C. glabrata* Carbonic Anhydrase CgNce103 enzyme may constitute a novel target for new classes of antifungals.

Carbonic anhydrases (CAs, EC 4.2.1.1) are a structurally diverse family of enzymes that catalyze the interconversion of carbondioxide (CO_2_) to bicarbonate (HCO_3_^−^). This reaction influences physiological pH values and the supply of HCO_3_^−^ ions and as such many physiological, metabolic, and biosynthetic pathways^6–14^. Inhibitors of these enzymes may constitute novel therapeutics against cancer or may have potential as antifungal drugs^6–16^. Recently, the inhibition of bacterial CAs by sulfonamide derivatives have been shown to inhibit the growth of pathogenic microorganisms^17–25^. CO_2_/HCO_3_^−^ equilibration by fungal β-CAs plays a critical role in CO_2_ sensing and as such is an important mediator of fungal metabolism and pathogenesis. One such enzyme is β-CA of the opportunistic pathogen *C. glabrata* (CgNce103). This enzyme has an important role in the CO_2_-sensing of the fungal pathogens^26–32^. CgNce103 is involved in the virulence and pathogenesis of *C. glabrata* due to its effects on the CO_2_/HCO_3_^−^ concentrations and as such it constitutes a novel target for antifungal agents with a novel mechanism of action^31–33^.

Supuran et al. have evaluated several sulfonamides, sulfamates, sulfamides *N*-mono, and *N,N*-disubstituted dithiocarbamates against CgNce103 in their search for structurally novel inhibitors^34–38^. The results indicated that several of their compounds are potent CgNce103 inhibitors^34^^,^^35^. As such, the development of selective and potent CgNce103 inhibitors may result in new antifungal drugs and could overcome the problem of developing resistance against currently used antifungal drugs.

In a recent study, we described the synthesis of novel 1*H*-indole-2,3-dione 3-thiosemicarbazone derivatives (compounds **4a**–**m**) carrying a sulfamoyl group at 4-position of the phenyl ring, molecular modeling studies for target enzymes and the effects of the synthesised compounds on tumor-associated hCA IX and XII enzymes^39^. All of the tested compounds showed selective inhibition in the low nanomolar range for hCA IX and XII enzymes over cytosolic isoforms hCA I and II enzymes. In this new study, these compounds were investigated for their inhibitions against *Candida* species CgNce103. Molecular modeling studies were conducted to rationalise the obtained inhibition values.

## 2. Experimental

### 2.1. CgNe103 enzyme inhibition assays

An Applied Photophysics (Leatherhead, UK) stopped-flow instrument has been used for assaying the CA-catalysed CO_2_ hydration activity^40^. Phenol red (at a concentration of 0.2 mM) has been used as indicator, working at the absorbance maximum of 557 nm, with 20 mM TRIS (pH 8.3) as buffer, and 20 mM Na_2_SO_4_ (for maintaining constant the ionic strength), following the initial rates of the CA-catalysed CO_2_ hydration reaction for a period of 10–100 s. The CO_2_ concentrations ranged from 1.7 to 17 mM for the determination of the kinetic parameters and inhibition constants. For each inhibitor, at least six traces of the initial 5–10% of the reaction have been used for determining the initial velocity. The uncatalysed rates were determined in the same manner and subtracted from the total observed rates. Stock solutions of inhibitor (0.1 mM) were prepared in distilled-deionised water and dilutions up to 0.01 nM were done thereafter with the assay buffer. Inhibitor and enzyme solutions were preincubated together for 15 min at room temperature prior to assay, in order to allow for the formation of the E–I complex. The inhibition constants were obtained by nonlinear least-squares methods using PRISM 3 and the Cheng-Prusoff equation, as reported earlier, and represent the mean from at least three different determinations^36^^,^^41–47^.

### 2.2. Molecular docking studies

Three-dimensional structures for compounds **4a–m** were generated in their lowest energy conformation (C=N double bond in Z isomer) using the MOE software package (v2018.0101, Chemical Computing Group Inc., Montreal, QC). The sulfonamide moiety was given a negative charge (R-SO_2_NH^−^), because this moiety binds to the active site Zn^2+^-ion. Subsequently, a steepest-descent energy minimisation protocol was applied using the MMFF94x force field. All ligands were docked into the active site of the CgNce103 homology model described in a previous study using the ChemScore scoring function (50 dockings per ligand; active site defined as all amino acids within 12 Å of centroid with coordinates *x*: 24.937, *y*: −20.463, *z*: −9.641) in the GOLD suite software package (v5.6.2, CCDC, Cambridge, UK)^36^.

## 3. Results and discussion

### 3.1. CgNce103 enzyme inhibition assays

Compounds **4a**–**m** and acetazolamide were tested in CgNce103 enzyme inhibition assays. The compounds inhibited CgNce103 with *K*_I_ values in the range of 6.4–63.9 nM (Table 1). Compounds **4g** (R = 5-I), **4h**, **4j**, **4k** and **4l** (R = 5,7-diCl) showed the best inhibitory effects against CgNce103 with *K*_I_ values 19.8, 19.8, 15.0, 12.8, and 6.4 nM, respectively. Among them, compound **4l** has the highest activity and the best selectivity for CgNce103 over hCA I and II. The *K*_I_ values of compound **4l** for hCA I and II are respectively 87-fold and 122-fold higher compared to CgNce103.

**Table 1.** CgNce103 enzyme inhibition data (*K*_I_, nM) for compounds **4a–m**.


Compound	R	hCA I*	hCA II*	CgNce103	hCA I/ CgNce103	hCA II/ CgNce103
**4a**	H	1190.0	936.0	63.9	18.6	14.6
**4b**	5-CH_3_	744.0	735.0	54.0	13.8	13.6
**4c**	5-OCF_3_	481.0	420.0	53.7	8.9	7.8
**4d**	5-F	558.0	551.0	34.7	16.1	15.9
**4e**	5-Cl	650.0	616.0	41.3	15.7	14.9
**4f**	5-Br	882.0	851.0	51.6	17.1	16.5
**4g**	5-I	624.2	399.4	19.8	31.5	20.2
**4h**	5-SO_3_Na	68.8	32.0	19.8	3.5	1.6
**4i**	5-NO_2_	901.0	897.0	42.1	21.4	21.3
**4j**	7-F	57.6	26.9	15.0	3.8	1.8
**4k**	7-Cl	76.1	425.6	12.8	5.9	33.2
**4l**	5,7-diCl	554.4	782.1	6.4	86.6	122.2
**4m**	5,7-diBr	698.0	687.0	46.6	15.0	14.7
**AAZ**		250.0	12.1	11.0	22.7	1.1

*K_I_ values were obtained from Ref. [39].

The unsubstituted compound **4a** showed the highest measured *K*_I_ value (*K*_I_: 63.9 nM), while the lowest *K*_I_ value was measured for the 5,7-dichloro substituted compound **4l** (*K*_I_: 6.4 nM). As such, there is only approximately 10-fold difference in the highest and lowest measured *K*_I_ value amongst compounds **4a**–**m**. This narrow activity window makes it rather difficult to suggest structure–activity relationships for these compounds. Together with the fact that the compounds only differ in their substituents on the 5 and 7 positions, we expect that the binding interactions of the compounds with the CgNce103 active site is very similar.

### 3.2. Molecular docking studies

Docking studies were performed to unravel putative ligand–enzyme binding interactions for this series of compounds. To this end, three-dimensional structures for compounds **4a–m** were generated in their lowest energy conformation (C=N double bond in Z isomer) and docked into the active site of the CgNce103 homology model as previously described^36^. In short, the crystal structure of *Saccharomyces cerevisiae* CA Nce103 (pdb code: 3eyx; 2.04 Å), which shows 52.3% sequence identity to CgNce103, was used as a template to construct the homology model for CgNce103 using the MOE software package^36^. The CgNce103 sequence was obtained from the National Center for Biotechnology Information (NCBI; GenBank: CAG59355.1; 219 amino acids). The template backbone was fixed during the homology model construction, The homology model with the highest contact score was selected and a steepest-descent energy minimisation protocol was applied using the AMBER12:EHT force field. To this end, all heavy atoms of the active site residues, the zinc ion, the zinc-binding residues, and the protein backbone were fixed and the other parts were minimised using a controlled release of position restraints. The minimised structure was used in the docking studies.

**Figure 1.**
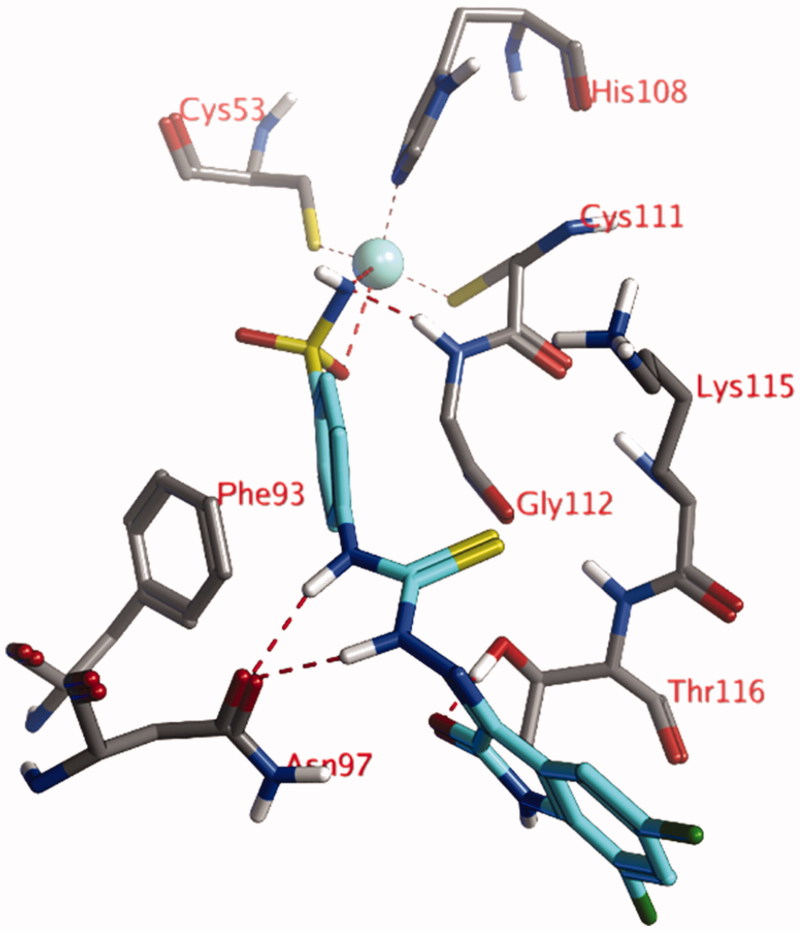
The docked pose of compound **4l** (turquoise) within the active site of CgNce103. Hydrogen bonds and interactions with the Zn(II) ion are indicated in red dashed lines.

The docked pose of compound **4l**, the compound with the lowest measured *K*_I_ value, shows an interaction of the anionic sulfonamide moiety (R-SO_2_NH^-^) with the active site zinc ion (Figure 1). The phenyl group adjacent to this sulfonamide moiety forms hydrophobic interactions (edge-to-face π–π stacking) with the aromatic side chain of Phe93. The thiosemicarbazone group forms hydrogen bonds with the side chain of Asn97, while the ligand’s carbonyl group forms a hydrogen bond to the side chain of Thr116. All other molecules of the tested series adopt very similar poses as described for compound **4l**, as the substituents points toward the solvent and do not form an interaction with the protein.

## 4. Conclusions

In this study, novel 1*H*-indole-2,3-dione 3-thiosemicarbazone derivatives **4a–m** carrying a sulfamoyl group at the 4-position of the phenyl ring were synthesised and tested against CgNce103 of the opportunistic pathogen *C. glabrata*. The compounds showed *K*_I_ values in the range 6.4–63.9 nM against CgNce103 and between 2-fold and 120-fold higher *K*_I_ values for the wide-spread human carbonic anhydrase isoforms I and II. Compound **4l** has the highest activity and the best selectivity for CgNce103 over hCA I and II. The selectivity rates of **4l** for CgNce103 over hCA I and II were found to be 4-fold and 111-fold higher than acetazolamide, respectively. Docking studies have suggested a possible binding pose for these compounds in the active site of CgNce103. These compounds may have the potential to serve as leads for developing new antifungal drugs with a novel mechanism of action which could overcome the problem of developing resistance against the classical antifungal agents.
